# Segmentation of Drilled Holes in Texture Wooden Furniture Panels Using Deep Neural Network

**DOI:** 10.3390/s21113633

**Published:** 2021-05-23

**Authors:** Rytis Augustauskas, Arūnas Lipnickas, Tadas Surgailis

**Affiliations:** 1 Department of Automation, Kaunas University of Technology, 51367 Kaunas, Lithuania; arunas.lipnickas@ktu.lt; 2 MB Prorega, 48212 Kaunas, Lithuania; tadas@prorega.lt

**Keywords:** CNN (convolutional neural networks), deep learning, image processing, hole detection, drilling, furniture manufacturing, quality inspection, industry 4.0

## Abstract

Drilling operations are an essential part of furniture from MDF laminated boards required for product assembly. Faults in the process might introduce adverse effects to the furniture. Inspection of the drilling quality can be challenging due to a big variety of board surface textures, dust, or woodchips in the manufacturing process, milling cutouts, and other kinds of defects. Intelligent computer vision methods can be engaged for global contextual analysis with local information attention for automated object detection and segmentation. In this paper, we propose blind and through drilled holes segmentation on textured wooden furniture panel images using the UNet encoder-decoder modifications enhanced with residual connections, atrous spatial pyramid pooling, squeeze and excitation module, and *CoordConv* layers for better segmentation performance. We show that even a lightweight architecture is capable to perform on a range of complex textures and is able to distinguish the holes drilling operations’ semantical information from the rest of the furniture board and conveyor context. The proposed model configurations yield better results in more complex cases with a not significant or small bump in processing time. Experimental results demonstrate that our best-proposed solution achieves a Dice score of up to 97.89% compared to the baseline U-Net model’s Dice score of 94.50%. Statistical, visual, and computational properties of each convolutional neural network architecture are addressed.

## 1. Introduction

Furniture manufacturing of laminated MDF (Medium-density fiberboard) panels is a multistage process that consists of many manual or automated steps. It starts with the production of the chipboard and its lamination. When the designed shape furniture panel is cut out, the milling and drilling process starts, which is the most crucial in furniture manufacturing. The arrangement of drilled holes is critical for successful final product assembly. Deviation from template corrupts the final product. The faults might appear due to various reasons: failures or misalignments in drilling machinery template set-up, wear-off or lose parts, dull or broken drill, and others. Moreover, the manual inspection itself requires a lot of time due to measurement evaluation and knowledge about the individual part template. The situation can get even more complicated due to different sizes of drillings, multiple holes (blind and through), different parts, scobs, and dust, and other defects. Therefore, this process needs to be automated.

Nowadays inspection of manufacturing processes is an essential part of industry 4.0 standards. Investigating the quality in each step of production might lead to detecting the flaws in early fabrication stages and reducing materials usage and operations time needed. In the end, manufacturing costs can be cut down. Besides the obvious results, the risk of defects appeared at sold production can be decreased as well. A non-invasive check-up, such as one that is computer vision (CV)-based, might be used in the most observable cases. As it is mentioned in the review [1], the visual-based approach for defect detection is one of the most common in the industry. It is complicated to take into consideration a variety of furniture board processing operations, possible defects, and complicated surface pattern cases. Moreover, the production quality evaluation factors can be disturbed by aggressive manufacturing conditions. However, from visual information, a broader context might be perceived. As mentioned before, the irrelevant parts, such as defects or milling, might appear along with drilled holes. Therefore, the algorithm should distinguish only the information that is pertinent for the task. Computer-vision-based methods need to act as an intelligent sensor for drilling localization.

In this paper, we present a novel, data-driven approach for contextual pixel-level drilled hole segmentation approach in textured wooden furniture panels from the images. We use a small architecture U-Net convolutional encoder-decoder network as a baseline and we are proposing the architectural modifications in a neural network with residual connections [2], atrous spatial pyramid pooling module [3], squeeze and excitation blocks [4], and *CoordConv* layers [5] that improves the standard architectures in Dice score for a pixel-level segmentation task with a slight computational performance increase. Besides the modification in models, we address segmentation precision and computational performance. We compare convolutional neural network results with conventional image processing methods to show the advantage of higher-level information representation and the ability to adapt to the context in a wide spectrum of cases. Our neural network implementation, conventional image processing comparison methods code, and more rendered results can be found in the GitHub repository [6].

## 2. Related Work

There can already be found computer-vision-aided approaches for through-hole inspection. In researches conducted by Hernandez et al. [7] and Caggiano et al. [8], the carbon fiber boards are being investigated. In papers, authors proposing segmentation based on Otsu threshold [9] and segmentation from HSV colormap respectively. Drilled hole contours can be separated, and the color/texture of boards is always constant. Another drilling inspection approach was described by Yu et al. [10]. Researchers have used multiple image preprocessing techniques and Canny edge [11] to extract holes and a flush for rivets in aircraft panels. More complicated hole segmentation in textured composites parts is presented in [12]. Authors were utilizing local binary patterns algorithm [13] in combination with deep learning segmentation with a lightweight U-Net convolutional neural network (CNN). Overall, the practical implementation of the proposed research on the drilled holes segmentation would be very limited. Most of the review articles are utilizing classical computer vision methods, such as thresholding (Otsu or from HSV colormap) or edge detection (Canny). Only one [12] of mentioned articles employs a deep neural network for more complicated hole image data.

Image processing algorithms can be a satisfying solution in a defined number of cases, however more dynamic inspection conditions or complex manufacturing processes or production require more advanced and higher-capability solutions. Representing a problem by strictly formed rules might be a narrow solution or it can get complicated to cover up states or situations in an extensive dataset. However, this problem can be overcome by utilizing data-driven solutions, such as deep learning (DL) approaches. Labeled samples can provide essential information for the chosen algorithm on how to cope with a particular task. Expert data knowledge and representation can be transferred to the model during its training process. Even extra-large-scale datasets, for example ImageNet classification [14], Microsoft COCO [15], and Open Images Dataset [16], are proven to be solved by engaging deep learning methods [17,18,19].

There can be found multiple application of artificial-intelligence-aided computer vision in a variety of automated manufacturing inspection cases, such as steel [20,21,22], wood [23,24,25,26], and resin/plastic [27,28,29]. The mentioned investigations utilize deep neural networks as an algorithm to distinguish defects. Taking into consideration segmentation of drilled holes in furniture panels problem complexity, data diversity and industrial environment working conditions there can be made relations with approaches used for defects detection and inspection methods used in manufacturing and severe environments.

In most cases, convolutional neural networks (CNN) are employed for image processing. Popular architecture-based solutions can be found in several papers. Gao et al. [23] proposes ResNet-34 [2] for wood knot detection and classification. Moreover, the authors are proposing transfer learning (using a pre-trained model backbone) to overcome a limited number of samples in the used dataset. Yin et al. [30] describe sewer pipes defects detection techniques from CCTV footage using YOLOv3 [31] object detection model. Another single-shot technique for printed circuit board abnormalities search is described by Adibhatla et al. [32]. Authors of solar panels manufacturing defects detection investigation [33] use Fast RCNN [34] with VGG-16 [35] backbone for defective regions search. Researchers also propose the Complementary attention network module for features extracted (from the backbone) feature refinement. Roberts et al. [36] applied U-Net [37] encoder-decoder with additional dense connection for crystallographic defects in steel images semantic segmentation. Additionally, from the following papers, it can be seen that even small architectures can perform well in defect identification tasks. In [21], researchers uses MobileNetV2 [38] backbone for welding classification. Modified versions with dense connections of mentioned architecture are proposed in [39] for DAGM [40] defective patterns classification. As authors suggest, introduced adjustment allows coping better with the multiscale problem. Even smaller convolutional design networks are described in steel wire defects detection investigation [22]. The solution utilizes three convolutional layers of neural network for a 3-class classification task. A similar approach (regarding small neural network architecture) can be found in wood defects detection and classification investigation [24]. A minimalistic convolutional neural network can be seen in resin defection research [27], where the LeNet-5-like model is being utilized. Moreover, light-weight segmentation approaches are investigated by Huang et al. [41], where only one step of upscaling is employed and another enhancement, atrous spatial pyramid pooling (ASPP) [3], is utilized. The proposed architecture solution has shown good results on DAGM [40], Wood defects [42], and NEU [43] datasets. It can be summed up that deep-learning-based computer vision can give a solution in complicated situations.

## 3. Methods

### 3.1. Convention Image Processing

There are multiple computer vision algorithms for feature extraction from visual information. Most of the statistical methods rely on local intensity differences in the data without contextual analysis. A classical method such as intensity threshold is more suitable for static data, which does not alter that much. However, “real-world” scenarios usually are not fixed in a particular way. Especially manufacturing environments tend to be more diverse in conditions and production visual complexity might vary. For the mentioned case there can be found more robust methods that are more adaptive to dynamic. For example, Sobel [44] or Laplace [45] filters signify changes in visual information intensities. Kernels of Sobel filter along *x* and *y* axes are given in Equations (1) and (2) and the kernel of the Laplace filter is given in Equation (3).

(1) $$G_{x}=\left[\begin{array}{ccc} 1 & 0 & -1\\ 2 & 0 & -2\\ 1 & 0 & -1 \end{array}\right],$$

(2) $$G_{y}=\left[\begin{array}{ccc} 1 & 2 & 1\\ 0 & 0 & 0\\ -1 & -2 & -1 \end{array}\right],$$

where $G_{x}$ and $G_{y}$ are Sobel filters kernel along *x*- and *y*-axes, respectively.

(3) $$D_{xy}^{2}=\left[\begin{array}{ccc} 0 & 1 & 0\\ 1 & -4 & 1\\ 0 & 1 & 0 \end{array}\right],$$

where $D_{xy}^{2}$ is the Laplace filter kernel.

Another popular and powerful method for edge segmentation is Canny edge detector [11]. Firstly, the algorithm utilizes Gaussian filter to reduce the noise in the image, after, abrupt intensity changes (possible edges) are extracted using Sobel filter along *x* and *y* axes. Subsequently, non-maximum suppression is applied to remove spurious edges and thresholding engaged to remove weak results. After that, edges are processed with hysteresis and small artifacts that are not connected to “strong” edges are removed. However, even with these methods, it can be hard to capture specific details when the context is sophisticated: a variety of possible color combinations, object surface with patterns, similarities between a significant (desired to extract), and a minor (background) information.

### 3.2. Baseline U-Net

**Figure 1:**
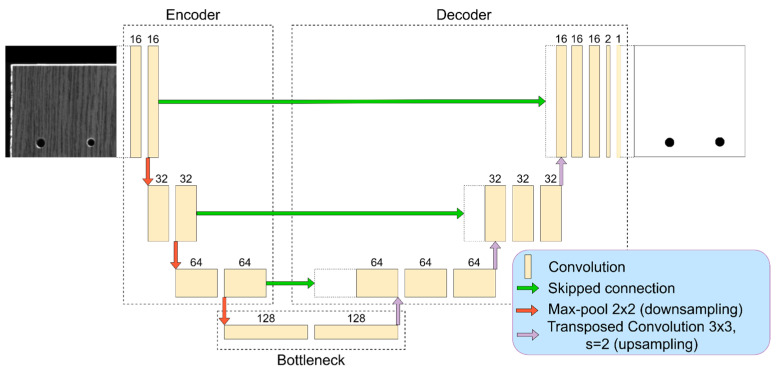
Baseline U-Net principal structure. The input is 320 × 320 px greyscale image and the output 320 × 320 px classified image (black is drilling segmentation, white is the background).

A more advanced segmentation approach—convolutional encoder-decoder (U-Net)—might be engaged. A data-driven model can represent features while taking into consideration not only the simple local intensity differences but also the relations between details and other semantical information. The knowledge presented in labels can be encoded into a high dimensional feature space and generalized making the U-Net a powerful tool for information extraction (segmentation) in a complicated context. In this work, as a baseline segmentation model, we employ a lightweight U-Net [37] convolutional neural network (Figure 1). The architecture consists of two main parts: encoder and decoder. The first extracts image features, and the second reconstructs the segmentation map. Opposite layers in the encoder and decoder are associated with skipped connections that allow transferring higher-level features from larger dimension layers. In this research, we utilized quite a small architectural design with three downscales. At the first stage (first layer) 16 feature maps are employed. After each width and height downscale by two, the number of feature maps is doubled. In the decoder, reversed operations are performed—dimensions upscale and feature maps count reduction by two. The output of convolutional encoder–decoder is 1 × 1 convolution with sigmoid activation which performs as binary classifier between two classes: drilled hole and background.

**Figure 2:**
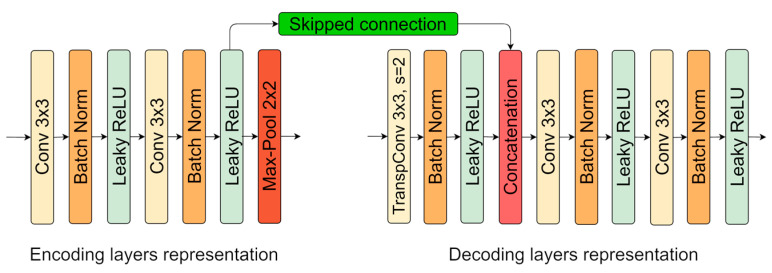
U-Net encoding and decoding layers representations.

A more detailed illustration of layers structure on opposite sides (encoder and decoder) is given in Figure 2. Each stage in the encoder consists of two convolutional operations with 3 × 3 size kernels, with a stride of 1 pixel. Only kernel size exception is applied in the first layer, where 5 × 5 is engaged. In decoder transposed convolution with 3 × 3 kernel and stride of 2 pixels. It increases the input dimensions by two. Further, it is a “learnable” approach for enlarged pixels interpolation. After upscale, feature maps from the previous layer are concatenated with opposite feature maps from the encoder (skipped connection). Every convolution and transposed convolution operation is followed by batch normalization [46]. It has trainable mean and variance parameters that help to keep output from convolution operation normalized. Moreover, it stabilizes the neural network model and increases training speed. As activation function, parametrized rectified linear unit or Leaky ReLU is operated. It is shown in the following equation:

(4) $$f\left(x\right)=\{\begin{array}{c} x,\;if\;x>0\\ 0.1x,\;otherwise \end{array},$$

where *x* is activation function input.

Additionally, we investigate the modified versions of U-Net. While an increased number of feature kernels in convolutional operation might end up in better segmentation results, it also loads a model with significantly more computational operations and prolongs execution time. We propose tricks and lightweight enhancements to improve segmentation efficiency while the impact on computational performance is not significant. Architectural changes are described more briefly in the following subsections.

### 3.3. Residual Connections

**Figure 3:**
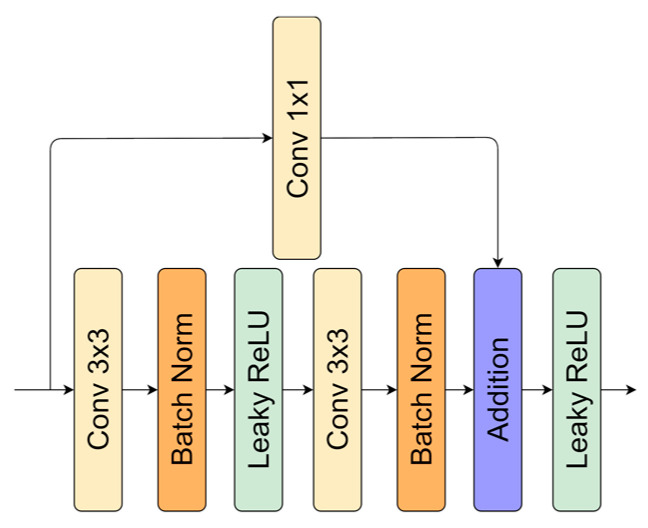
Residual layer representation.

Residual layers are proposed in ResNet [2]. The branch connected in parallel skips convolutional operation. Residual connections help to maintain information flow through the whole network, without a possible degradation in series of operations conducted in a neural network. Moreover, this block increases model accuracy and might cope with the vanishing gradient problem. Residual layers are used in popular architectures, such as SqueezeNext [47], DeepLab [48], and Inception [49]. The implementation used in this research is shown in Figure 3. We utilize 1 × 1 convolution to make the number of feature maps the same before the addition operation.

### 3.4. Squeeze-and-Excitation

**Figure 4:**
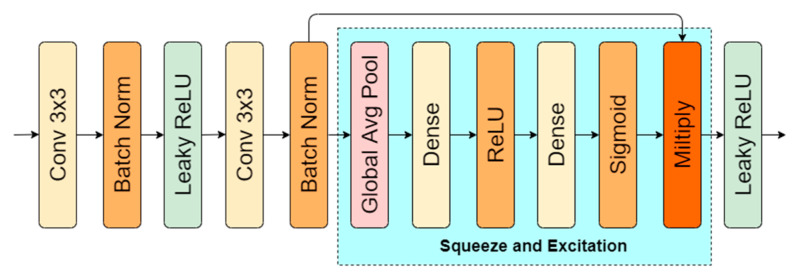
Squeeze and excitation block representation.

A light-weight solution proposed by Hu et al. [4] adaptively adjusts individual feature map weight. Squeeze and excitation (SE) block average each feature map to trainable fully connected neuron layers (Figure 4). After the second layer, sigmoid activation is applied that outputs values in the range [0.0,1.0]. Each value is a scalar for each feature map matrix. They recalibrate the significance channel-wise, taking into consideration dependencies between feature maps. In the mentioned research [4], squeeze and excitation enhanced convolutional neural network shown image classification accuracy boost on ImageNet [14], while not adding a lot computations to model (ResNet-50–top-1 error 24.8% (3.86 GFLOPs), ResNet-50-SE–top-1 error 23.29% (3.87 GFLOPs)).

### 3.5. Atrous Spatial Pyramid Pooling

**Figure 5:**
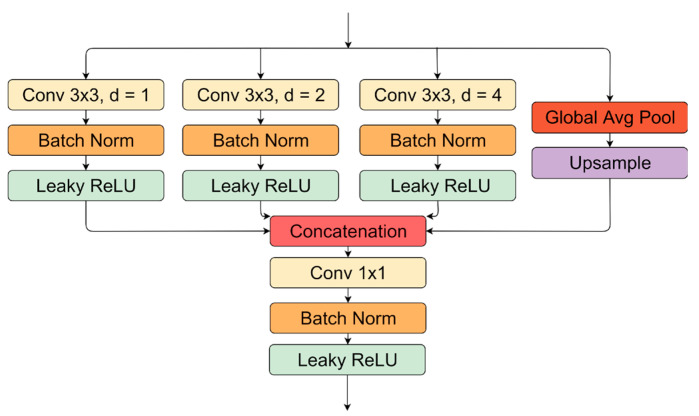
Atrous spatial pyramid pooling module.

Convolutional operations with different dilation rates might extract multi-scale contextual information better than regular convolutions (with a dilated rate equal to 1). Atrous or dilated convolutions in the parallel idea was proposed by Chen et al. [3]. An expanded convolutional kernel can better respond to different resolution features. In our research, we used three parallel branches with three different dilation rates: 1, 2, and 4 (Figure 5). However, some papers utilize bigger rates. Even in the previously mentioned research, the authors used 6, 12, and 18 dilation rates in convolutional kernels. In another research [50], the authors conducted multiple experiments with various rates, which yielded different results. Our dilation rates were chosen with the motivation of not severe changes in the data view scale. Additionally, we added another branch in parallel with the average pooling of individual feature maps and upscaling to capture global information in the particular feature channel. This idea is inspired by ParseNet [51] approach.

### 3.6. CoordConv

**Figure 6:**
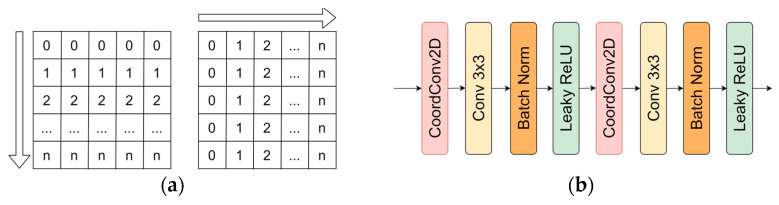
*CoordConv2D* representation: (**a**) 2 feature maps along *x*- and *y*-axis, indicating the position of the pixel at each axis; (**b**) *CoordConv2D* implementation before convolutional operation (adds 2 features maps—y-axis rows and x-axis columns indices).

An interesting approach by encoding position coordinates to cope with the data transition invariance problem was proposed by Liu et al. [5]. The authors suggested an idea to boost the prediction performance by introducing additional information in feature maps. *CoordConv* practices in convolutional neural networks have shown improvements in prediction [52,53,54]. For two-dimensional information, the authors propose two additional channels with a row index along the *y*-axis and a column index along the *x*-axis (Figure 6a). In this research, *CoordConv* operation is joined with other feature maps before convolutional operations (Figure 6b).

## 4. Data

### 4.1. Image Capture Setup

**Figure 7:**
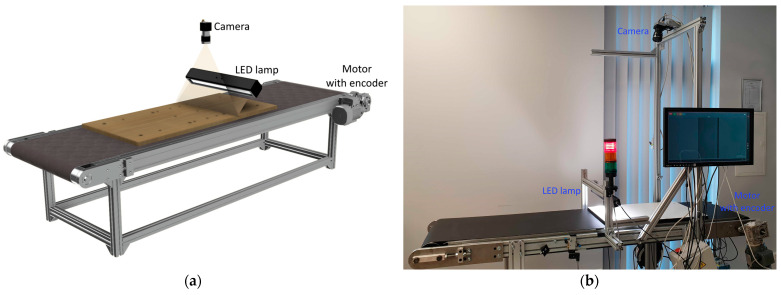
The image capturing set: (**a**) principle scheme, (**b**) laboratory setup. Linear camera pointing into the same line (area) as an industrial LED light source. The camera is triggered by an encoder on the motor.

Wooden furniture panels are scanned with a linear camera from an industrial conveyor. The image data acquisition stand (laboratory) is shown in Figure 7. The main parts of the visual inspection setup are a linear monochromatic camera with a scan width of up to 6144 pixels, an industrial LED light source, and a conveyor. The camera is attached 1.1 m above the conveyor belt. Its capture area (line) collides with an industrial LED light directional normal at the same line. Only the area around the camera scanning line is illuminated at a particular moment. Furniture panels are moved by a conveyor belt driven by the electrical motor. This motor is equipped with an encoder that triggers a scan of the linear camera. The start of capturing is invoked by a separate laser sensor that gives a high output signal when the furniture panel approaches the scanning area. The image capturing continues until the laser sensor signal is high or until the set image height is scanned. The mentioned links result in the system synchronization-camera scanning is triggered according to conveyor rotation (start and continues line scan). The equipment used for data grabbing is given in Table 1. The image capture setup is separate from the whole production line. Before furniture panels reach the visual inspection conveyor, they are directed by correcting the alignment. The object on the conveyor is always perpendicular to the scanning line. The physical orientation error does not exceed the 2° angle.

### 4.2. Wooden Furniture Panels Image Data

**Figure 8:**
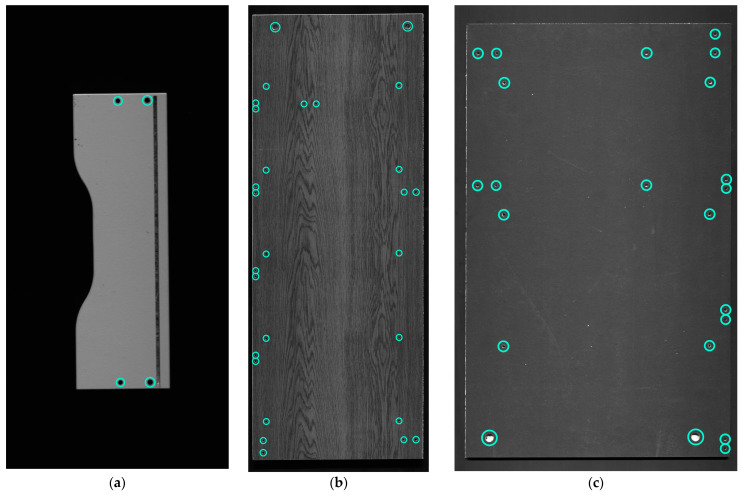
Data example. (**a**–**c**) different furniture panels in production: (**a**) part with cutout, (**b**) panel with wood pattern imitation, (**c**) black panel.

The size of furniture panels varies significantly. Depending on the manufactured product, the dimensions of the part can be as small as 0.13 m × 0.4 m (front panel of a table drawer) and as big as 0.9 m × 2.0 m (side of a cupboard) (Figure 8a–c). While there is a big diversity between panels size, there is no need to constrain the image size to be the same for all parts. Smaller furniture panels do not occupy the whole scanning area and it is pointless to analyze the rest of the conveyor context (outside the furniture part boundaries). As the information is not relevant for the analysis, the scanning range in width (as well as height) is adjusted. The image dimensions used in this research vary in width from 1000 to 6144 and height from 900 to 12,384 pixels. The biggest (consisting of two joined frames) image is 6144 × 12,384 pixels.

Additionally, to the furniture panels’ dimension and image size variety, there are big alterations in production exterior texture and colors. A few samples can be seen in Figure 8a—white, Figure 8b—wood pattern imitation, Figure 8c—black. For better details extraction and enhancement, different exposure rates are set for image capture. It ranges from 100 to 500 nanoseconds. In the case of white laminate on the furniture panel (Figure 8a), a smaller value can be applied. Hole and cutouts made by drilling or milling are easier to distinguish from the rest of a board context. Nonetheless, it gets complicated on the other samples (Figure 8b,c). In the darker color furniture panels, it is harder to extract details with a lower exposure rate. However, increasing this parameter strengthens other non-desirable details, such as the visible bottom of the drilling (light and dark wooden pattern), dust, prints on the furniture panel. In addition, manufacturing defects might appear, the drilled hole might be covered with woodchips, or surface laminate might be ripped up. Moreover, one side of the drilled hole might get more illuminated than the other (Figure 8b,c), and also cutouts might be made in particular parts.

Overall, there is a great change in conditions: furniture panels dimensions, appearance, visual defects, and cutouts. These factors are taken into consideration for the unified drilled hole segmentation solution.

### 4.3. Data Preparation

**Figure 9:**
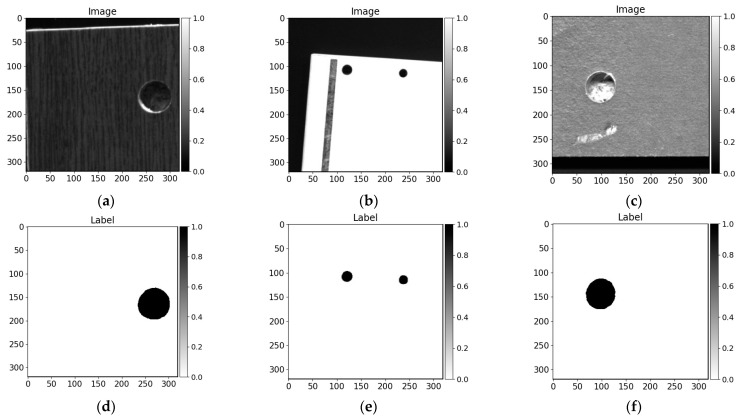
Prepared (cropped) data samples of 320 × 320 px size. All images are scaled to the range [0,1]. (**a**–**c**) cropped images, (**d**–**f**) drilled holed annotations.

In this research, we utilized a variety of images. As is mentioned in the previous subsection, the dimensions of data samples are changing severely. Further, most of the pictures are extremely large—exceeding 72 megapixels. Moreover, only the board context is useful for possible hole drilling segmentation. Taking into consideration the hardware limitation with model resources in video memory on the graphical processing unit (GPU), the dimensions of the data sample fed to the convolutional neural network cannot be relatively large. We utilize the tiles technique when the whole image is cropped into desired size regions with overlap. In this investigation, we divided the picture into 320 pixels width and 320 pixels height regions with 80 pixels overlap. A few samples (image and label) are shown in Figure 9. In this research, we also used not positioned data (not perpendicular to camera scan line as it is mentioned in the previous subsection) because parts of training data are grabbed by placing furniture panels on the conveyor by hand while skipping the orientation adjusting step (Figure 9b).

**Figure 10:**
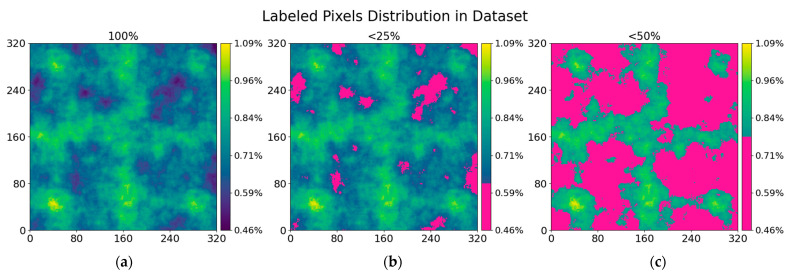
Individual pixels overlap by the label in the augmented dataset. (**a**) Full coverage of individual pixel covered by hole label pixel. (**b**,**c**) Areas where pixels are covered less: **b** < 25%, **c** < 50% in comparison with the maximum covered area (1.09% of data samples).

Before image crop to tiles, we augmented the picture by rotating by 90° four times, resizing to 90% and 110% of original sample size, random brightness correction in the range [−10;10] (considering image intensity range [0;255]), random Gaussian noise and gamma correction. In this research, we used 189 images divided into 151 for training and 38 for testing. The drilled hole area is relatively small compared with the rest of the background. As the result, there might be not a lot of positive samples. We considered it randomly (with a 50% possibility) taking out region tiles that do not contain marked hole pixels. Moreover, regions with an average intensity of 5 and less there added only with a 10% possibility. These regions are conveyor belt regions, that occupied a lot of area in the picture with small furniture panel. By reducing negative samples, we increase the size of more contextually essential data—regions with drilled holes. The augmented training dataset contained 86,180 grayscale 320 × 320 pixels images and annotations. Labeled holes’ pixels distribution through the image can be seen in Figure 10. Every place in the prepared data is covered at least in 0.46% of samples and the maximum covered area is in 1.09% of samples. More signified places of hole labels are given in Figure 10b,c. The most annotated regions in the augmented dataset are near corners and along vertical and horizontal center lines.

## 5. Experiments and Evaluation

The convolutional neural network architectures were written in Python (v3.7.9) using Keras abstraction layer [59] on Tensorflow 2.4.0 [60] backend. Experiments were conducted on desktop and laptop computers with parameters given in Table 2. Model training and testing were done in Windows 10 environment. Models trained on the desktop computer.

In this paper, specific modification’s influence on prediction precision and computational performance are investigated. We trained and analyzed eight different convolutional encoder–decoder architectures:

- UNet;
- UNet with a squeeze and excitation (*UNet + SE*);
- UNet with CoordConv (*UNet + CoordConv*);
- UNet with a squeeze and excitation and CoordConv (*UNet + SE + CoordConv*);
- UNet with residual connections and atrous spatial pyramid pooling (*UNet + Res + ASPP*);
- UNet with residual connections, atrous spatial pyramid pooling, and squeeze and excitation (*UNet + Res + ASPP + SE*);
- UNet with residual connections, atrous spatial pyramid pooling, and CoordConv (*UNet + Res + ASPP + CoordConv*);
- UNet with residual connection, atrous spatial pyramid pooling, squeeze and excitation, and CoordConv (*UNet + Res + ASPP + SE + CoordConv*).

We chose a combined loss function consisting of cross-entropy (Equation (5)) and *Dice* loss (*Dice* score—Equation (6), and *Dice* loss—Equation (7)). The first part, cross-entropy, is quite often used loss function that describes the likelihood or probability distribution between two sets. By default, it can be found in popular machine learning frameworks. Cross-entropy loss is **X** value related to **Ẋ** value in the following expression:

(5) $$L_{CE}=-\frac{\sum_{i=1}^{N}x_{i}\cdot \log\left({\dot{x}}_{i}\right)}{N},$$

where $L_{CE}$—cross-entropy loss, $x_{i}$—*i* pixel value in label matrix **X**, ${\dot{x}}_{i}$—*i* pixel value in neural network prediction matrix **Ẋ**, and *N*—a total number of pixels.

The second loss function is *Dice* [61] loss. *Dice* loss evaluates the similarity of two datasets by overlap that is measured in the range from 0.0 to 1.0. In image segmentation, *Dice* score describes the overlap of sets—label and prediction.

(6) $$D_{score}=\frac{2\cdot \left|X\;\cap \;\overset{\cdot }{X}\right|}{\left|X\right|+\left|\overset{\cdot }{X}\right|},$$

(7) $$L_{D}=1-D_{score},$$

where $D_{score}$—*Dice* score, X—label matrix, **Ẋ**—predicted matrix, $L_{D}$—*Dice* loss.

The loss function used in this research is expressed in the following Equation (8):

(8) $$L=0.5L_{D}+0.5L_{CE},$$

where L—loss function, $L_{D}$—*Dice* loss, $L_{CE}$—cross-entropy loss.

Each convolutional neural network architecture trained for 15 epochs, by reducing the learning rate by half every 3 epochs (scheduled reduction). Starting rate was set to be 0.001. Adam optimizer [62] was employed in the training process. The data mini-batch was set to eight samples. The whole dataset (86,180 augmented regions images) is covered by 10,770 steps/iterations in every epoch. The model is tested at the end of every epoch. The evaluation was conducted on 38 test images dividing them into 320 × 320 pixel regions (same as training data) with 160 pixels overlap. The best performing solution (according to *Dice* score) from every training has been evaluated with the *Accuracy*, *Recall*, *Precision*, *Dice* score (same Formula (12) can be expressed as in Equation (6)) and *IoU* measures:

(9) $$Accuracy=\frac{TP+TN}{TP+TN+FP+FN},$$

(10) $$Recall=\frac{TP}{TP+FN},$$

(11) $$Precision=\frac{TP}{TP+FP},$$

(12) $$D_{score}=\frac{2*Precision*Recall}{Precision+Recall},$$

(13) $$IoU=\frac{GroundTruth\;\cap^{\;}\;Prediction}{GroundTruth\;\cup^{\;}\;Prediction},$$

where *TP* is true positive (correspond to correct detection of pixels that belong to the labeled defect area), *TN* is true negative (are the non-defective “background” pixels that are correctly recognized by the detector), *FP* is false positive (are wrongly detected defect pixels), *FN* is false negative (are the defect pixels that have been undetected by the detector), *GroundTruth* are labeled image pixels. The *Precision* measure indicates the proportion of false alarms; the *Recall* refers to the proportion of not detected defect pixels; and $D_{score}$ is *Dice* score or harmonic mean of *Precision* and *Recall*.

## 6. Results

### 6.1. Conventional Image Processing Methods

**Figure 11:**
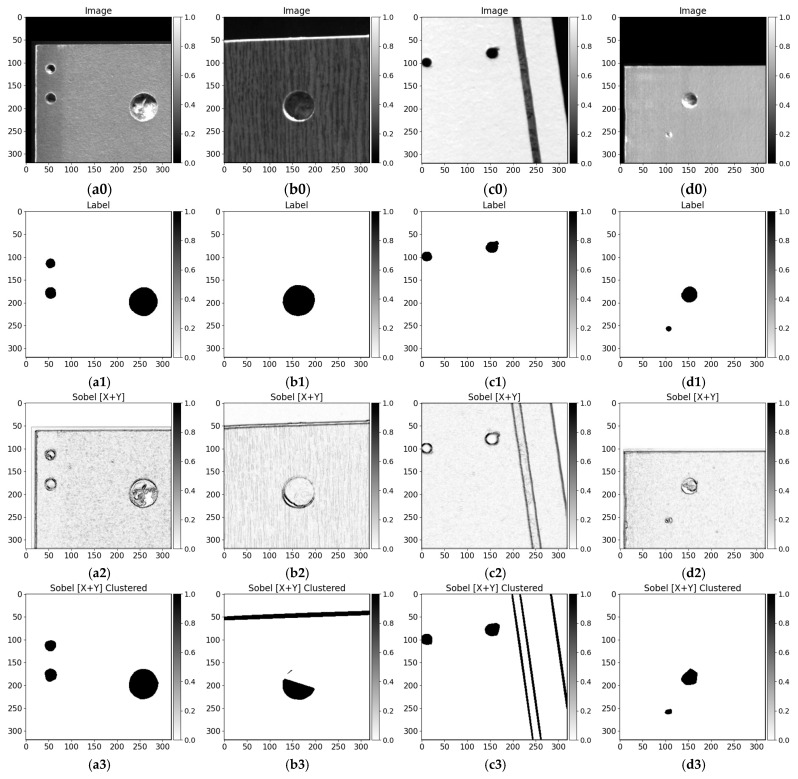

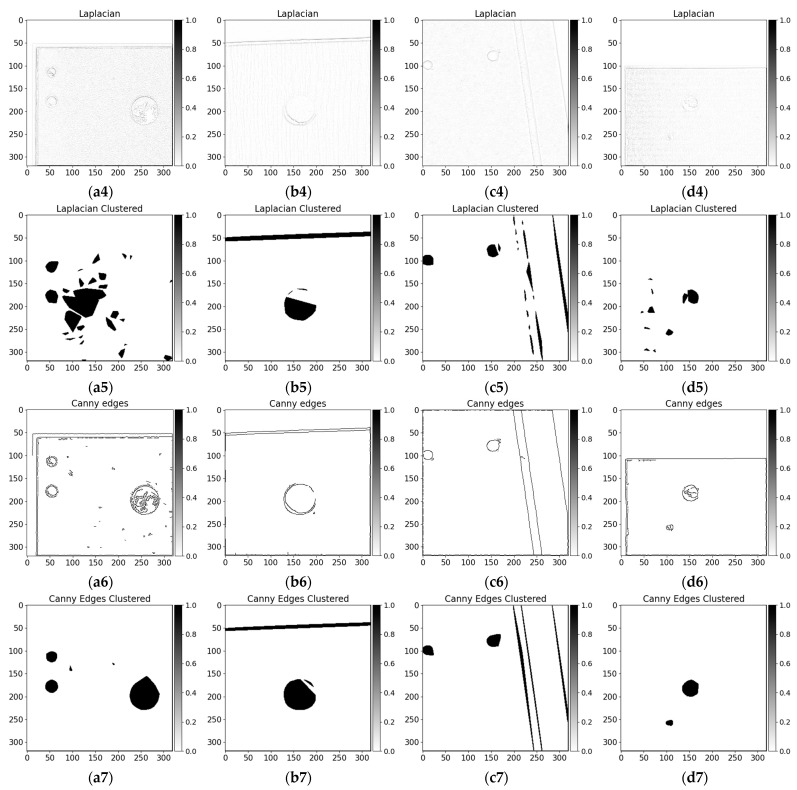
Sobel (3 × 3 filter, along X and Y axes), Laplace filter, and Canny edge detector results. Images—**a0**–**d0**, labels—**a1**–**d1**, Sobel filter results—**a2**–**d2,** Sobel filter clustered results—**a3**–**d3**, Laplace filter results—**a4**–**d4**, Laplace filter clustered results**—a5**–**d5,** Canny edge detector results—**a6**–**d6,** Canny edge detector clustered results—**a7**–**d7**.

In contrast to data-driven approaches using a convolutional encoder–decoder, we also compared traditional image processing methods results on drilled furniture data. We tested furniture board images with different surface patterns with Sobel filter (3 × 3 along X and Y axes), Laplace filter, and Canny edge detector. Visual results are given in Figure 11. It can be seen that filtering by local intensity tends to extract the edges (higher difference in neighbor pixel values). Sobel filter (Figure 11a2–d2) segments the boundaries the best among all compared conventional methods although it gets harder to distinguish the changes when the surface is complicated (Figure 11b2). Further, the Sobel filter is prone to reacting to the surface patterns even when these are insignificantly changing in surface colors. Moreover, in the case where drilled hole (blind) bottom is illuminated (Figure 11a2,d2), the transitions between wood chips are signified even more. The images processed with the Laplace filter (Figure 11a4–d4) give weaker features of edges after drilling and milling. It gets hard to distinguish the boundaries in the images shown in Figure 11a4,d4. Canny edge detector produces visually defined hole drilling edges, although some of them are not entirely closed or inside the drilling method tends to extract the pattern differences in wood chips of fiberboard (Figure 11a6,d6). Sobel, Laplace, and Canny edge filter segmented the milling gap shown in Figure 11c0. All conventional methods signify the differences in any pixel intensity changes. They do not carry out the ability to represent higher-level information or needs an additional step to perform data filtering. Moreover, methods tend to react to the pattern and require post-processing to finalize output prediction. We thresholded 50% of max processed (with filter) image intensity and clustered [63] edges points with a max distance of 5 pixels between neighbor pixels. Each cluster was closed with a convex hull [64], because edges tend to be open. Moreover, too small (5 pixels area) and too big (more than 20% of image 320 × 320 size) were filtered out. Post-processed results of Sobel filter are shown in Figure 11a3–d3, Laplace filter in Figure 11a5–d5, and Canny edge detector in Figure 11a7–d7. Even after edges contours clusterization, and additional filters, it is hard to define the drilling boundaries. The drilled hole shown in Figure 11b0 is being not fully extracted by all image processing algorithms and Laplace filter and Canny edge detector algorithm is tends to react to surface noise in Figure 11a5,a7 respectively. All methods extracted cutout from Figure 11c0 and board edge from Figure 11b0,c0. Due to the maximum size contour, filter edges from Figure 11a0,d0 are filtered out. The performance results of discussed image processing algorithms are given in Table 3. Considering a small hole area in the image (as shown in data overlay maps in the image in Figure 10), algorithms yielded high accuracy because the most of background predicted correctly. However, *Precision*, *IoU*, and *Dice* scores reveal that the performance of drilling segmentation is not as high. Along with investigated image processing algorithms, it can be seen that the Canny edge detected performs best.

**Figure 12:**
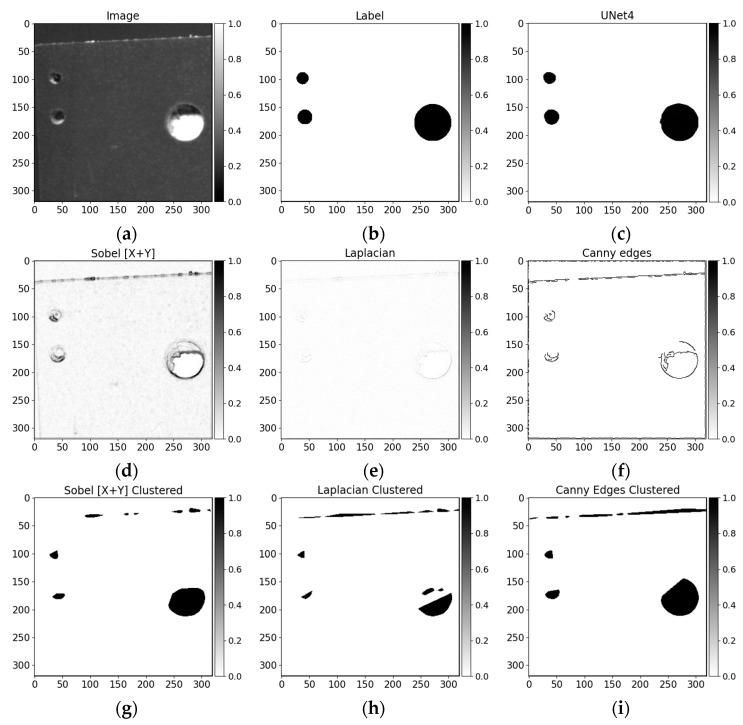
(**a**) Image, (**b**) label, (**c**) U-Net prediction, (**d**) Sobel filter, (**e**) Laplace filter, (**f**) Canny edges detector, (**g**) Sobel filter clustered, (**h**) Laplace filter clustered, (**i**) Canny edges detector clustered.

The edge cases where conventional methods fail to deliver satisfactory results can be seen in cases with a darker board surface pattern (Figure 12d–i filtered and clustered). Comparing with U-Net convolutional neural networks produced results (Figure 12c), it can be seen the differences between the data-driven and traditional methods capabilities in data dynamics. Even the shallow baseline U-Net model architecture captures the context with lightning variations (taking into consideration the precision around drilling edges).

### 6.2. Convolutional Neural Network Results

Each convolutional neural network architecture’s best-performing weights are picked according to the best *Dice* score on the test dataset. The results are given in Table 4. Any additional block to “baseline” *UNet* increased most of the overall results. A minimal 0.8504% increase in the *Dice* score can be seen by only enhancing the model with the squeeze and excitation blocks (*UNet + SE*). A more noticeable score increase can be seen by any other (*CoordConv, Res + ASPP*, etc.) addition to the original *UNet* model. The biggest *Dice* score is produced by encoder-decoder architecture with residual connections, atrous spatial pyramid pooling module, squeeze and excitation blocks (*UNet + Res + ASPP + SE*). It surpasses “baseline” by 3.3905% (in *Dice* score). Moreover, the particular solution yielded the highest *Recall* score. The top result in precision is produced by *UNet* with squeeze and excitation and *CoordConv* (*UNet + SE + CoordConv2D*). The same solution gave the highest intersection over union (*IoU*) score. Comprehensively, the accuracy measurement in this data case is not relevant, because it does not reflect the actual prediction performance accurately properly label-wise. The hole annotation is small and it takes a relatively small area compared with the background. The true negatives (*TN* is the right prediction on the background) make the biggest influence area-wise on the overall *Accuracy*, while the true positive (*TP* is right-predicted drilled hole pixels) might not make a significant impact on the score. This can also be seen in Table 4, where the differences in *Accuracy* measurements along models are indistinguishable and severely saturated due to precise predictions along most of the image context.

**Figure 13:**
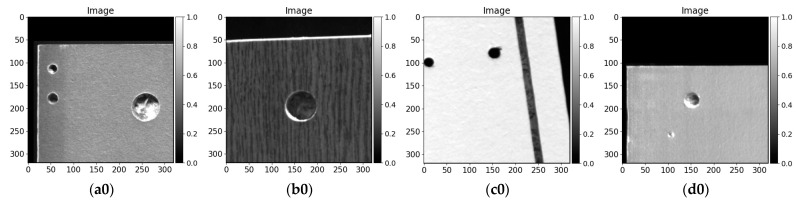

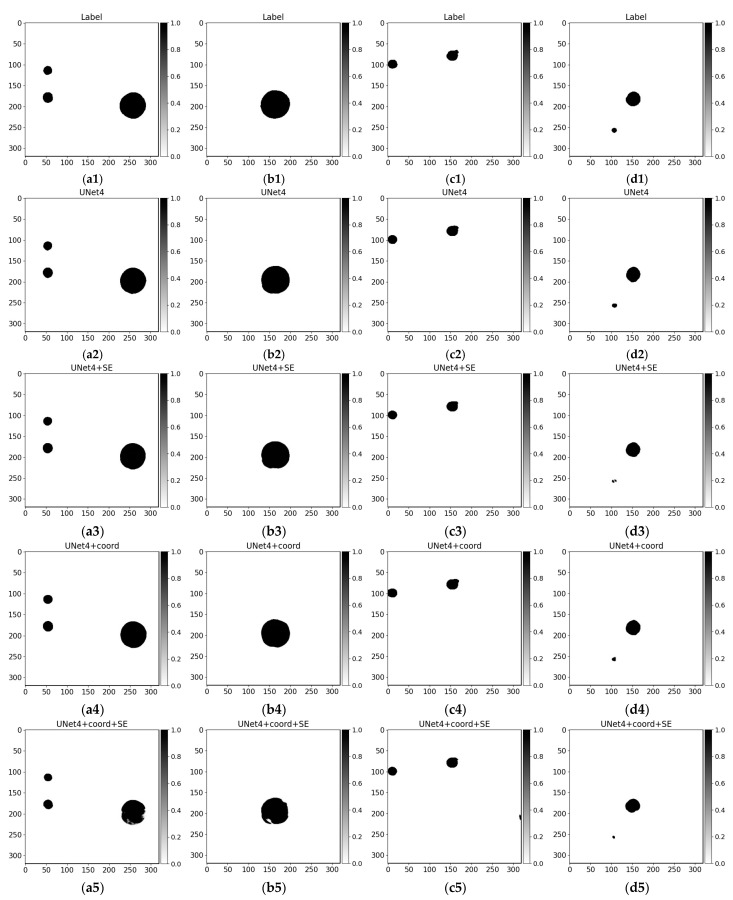

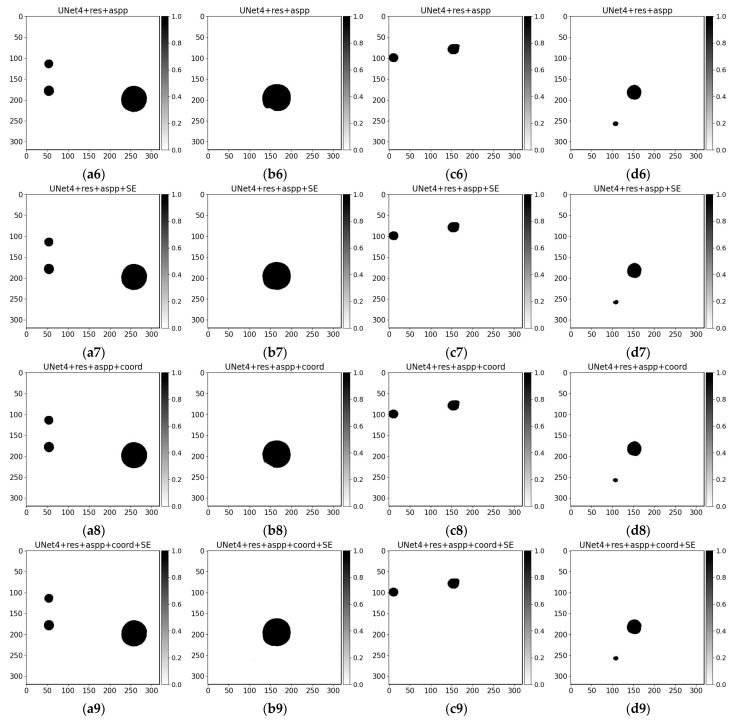
Each neural network architecture output on 4 cropped samples from the test set. Images (**a0**–**d0**), labels (**a1**–**d1**), network predictions (**a2**–**d9**).

Each model’s output on four different test set samples is given in Figure 13. We show results on the same data samples processed by conventional image processing methods (Figure 11). All architectures performing well on more common drilling samples, such as the left side of Figure 13a0 or the left side of 13c0. Moreover, all models are able to detect holes and separate them from another furniture panel processing, the milling cutout (Figure 13c0), despite the same wood chip pattern below the surface lamination. In drilling segmentation, even baseline *UNet* delivers visually appropriate results. Although, according to *Precision* (Table 4), the architecture yields more false-alarm predictions. The difference between convolutional neural networks might be more significant around the drilled hole edges and in more arduous samples. Figure 13a0,b0 have wider drilled holes. Additionally, there are drilled holes sides that are contrary illuminated—the lower part is more saturated. Sample in Figure 13a0 is handled better; however, *UNet + SE + CoordConv2D* is not as capable to segment the right side of the drilled hole (Figure 13a5). The same solution produces a small gap in Figure 13b5. Slight variations in prediction output can be seen between *UNet + SE* (Figure 13b3), *UNet + RES + ASPP* (Figure 13b6) and *UNet + RES + ASPP + CoordConv* (Figure 13b8) around the lower saturated hole edge—dilated or eroded edge. A rarer case with shallow drilling is given in Figure 13d0. A smaller diameter hole is entirely lit up and also the bottom part of the drilling might be similar to the top lamination (color- and texture-wise). Models enhanced with residual connections and atrous spatial pyramid pooling are able to capture the bigger context of the drilling. Interestingly enough, even “baseline” *UNet* segments a similar area of the hole. However, mentioned model’s drawback can be highlighted on the same image (Figure 13d0) centered drilling. In Figure 13d2, the “visual roundness” of the extraction is not as good as from models with *RES* and *ASPP*. However, architecture configuration *CoordConv* and squeeze and excitation modules (Figure 13d5) yields even worse output.

**Figure 14:**
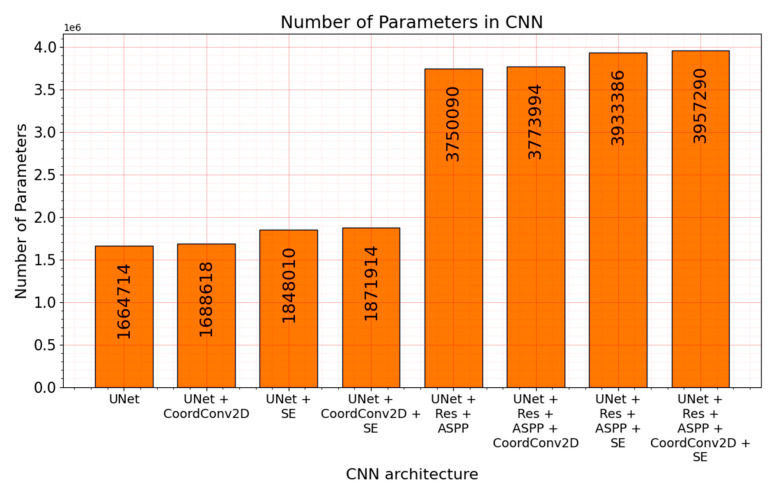
Number of parameters in each convolutional encoder–decoder.

Despite the models’ output precision benchmarks and visual evaluation, the computational performance aspect needs to be taken into consideration. Prediction speed is also critical in the best solution selection because the drilling visual analysis time is limited. The tradeoff between the speed and precision needs to be taken into comparison. While there are a lot of enhancements to the “baseline” *UNet*, there can be a noticeable increase in parameters. As it is given in Figure 14, solutions with residual connections and atrous spatial pyramid pooling modules double the number of neural network parameters. The minimal difference can be seen in architectures with *CoordConv* and slightly bigger in modifications with the squeeze and excitation blocks.

**Figure 15:**
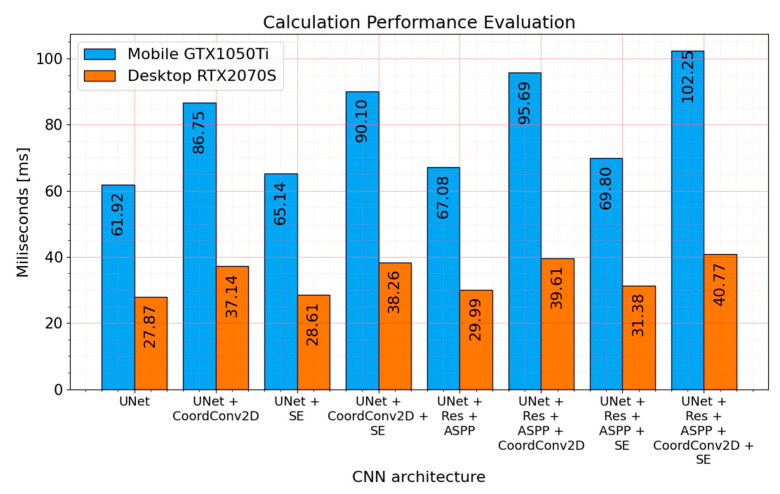
Each convolutional encoder–decoder performance prediction speed on Nvidia GTX1050Ti mobile (laptop) and Nvidia RTX2070 Super (desktop) GPUs. Tensorflow 2.4.0 prebuild from Python PIP package manager is used. Each time is averaged from 1000 forward image passes through the individual model.

However, the number of parameters does not directly correlate with computational speed. As it can be seen in Figure 15, the architectures with the biggest number of parameters (enhanced with residual connections and atrous spatial pyramid pooling) are not increasing prediction time significantly.

Comparing *UNet* and *UNet + RES + ASPP* computational speed, there is only a 7.61% increase in the system with Nvidia RTX 2070 Super and 8.33% in Nvidia GTX 1050 Ti laptop GPU. Even smaller prediction time increases can be seen in solution with a squeeze and excitation blocks (*UNet + SE*)—2.66% and 5.20% in desktop and laptop GPUs, respectively. In this particular case, computational speed decrease is more noticeable in mobile GTX 1050Ti. The biggest prediction time bump is noticeable in every solution with *CoordConv*—33.26% and 40.10%, respectively, in desktop and laptop machines. However, it is not a native Tensorflow 2.4.0 library layer and the results might be improved. Further, the speed might vary on implementation. The best-performing solution according to *Dice* score (*UNet + RES + ASPP + SE*) takes 12.59% and 12.73% more time or 3.51 and 7.88 milliseconds longer, respectively, in investigated desktop and laptop computers. The time for multiple image processing can be reduced by passing multiple images at once. For example, processing of input consisting of 16 images (shape (16, 320, 320, 1)) took 148.11ms on RTX2070S with *UNet + Res + ASPP + SE* model.

## 7. Discussion

In this work, we proposed a computer-vision-based approach for drilled blind and through-hole segmentation in wood chip furniture panels using convolutional neural networks. We also conducted experiments with *Sobel, Laplace* filters and *Canny* edge detector for comparison. The conventional image processing methods tend to segment simple samples; however, even with post-processing and edges filtering it was hard to fully distinguish the edges of the drilling in complicated cases. Moreover, methods reacted to the intensity differences on the board edges and complicated board surfaces. The best performing solution with image processing—*Canny* edge detector produced a 0.685342 *Dice* score, which significantly fell behind the baseline UNet solution with 0.944966.

On the samples containing a large variety of different surface lamination textures, milling cuts, and other faults appearing in the production, deep-learning-based models performed well. It was shown that despite the complexity in images, even a lightweight UNet model is able to generalize and segment drilled holes. This research revealed that more advanced modules and layers increased the model’s segmentation accuracy. Differences might be more distinguishable in more complicated samples. As the main subject of the investigation, UNet architecture was enhanced with squeeze and excitation block, *CoordConv* layers, residual connections, and atrous spatial pyramid pooling modules and inspected in segmentation and computational performance. All proposed model architectures with modifications yield results with a higher *Dice* score, compared with “baseline” architecture. Neural network models induced with squeeze and excitation (*UNet + SE*) raised *Dice* results by the minimum 0.8504%, while significantly better composition with *CoordConv* (*UNet + CoordConv*) boosted by 2.1009%. However, the combination of these two mentioned modules with “baseline” UNet (*UNet + SE + CoordConv*) did not give a better solution. From the images, it can be seen that it outputs significantly worse results in more rare cases. The best-proposed neural network configuration employed in this research was UNet with residual connection, atrous spatial pyramid pooling, and squeeze and excitation blocks (*UNet + RES + ASPP + SE*). It increased *Dice* score by 3.3905% (comparing with “baseline” UNet), scoring 0.978871 on 320 × 320 pixel image in 31.38 and 69.8 milliseconds (taking 3.51 and 7.88 milliseconds more than “baseline” solution) on desktop RTX 2070S and laptop GTX 1050Ti. Enhancing this architecture with *CoordConv* resulted in poorer segmentation. Moreover, in all cases, the mentioned layer resulted in a significantly bigger computation time −33.26% and 40.10% in desktop and laptop systems, respectively, comparing to the base model. On the other hand, it was the custom *CoordConv* implementation that was not a part of the deep learning framework. The overall time of analysis can be reduced by passing bigger input formed from multiple images to the model. The input of 16 images (16, 320, 320, 1) took 148.11ms to process and the input of one image (1, 320, 320, 1) took 31.38ms on RTX2070S with *UNet + RES + ASPP + SE* model.

The proposed neural network model or modifications can be engaged in problems such as remote image segmentation [65,66], medicine [67,68], faults detection [69,70], and others.

## 8. Integration and Future Work

**Figure 16:**
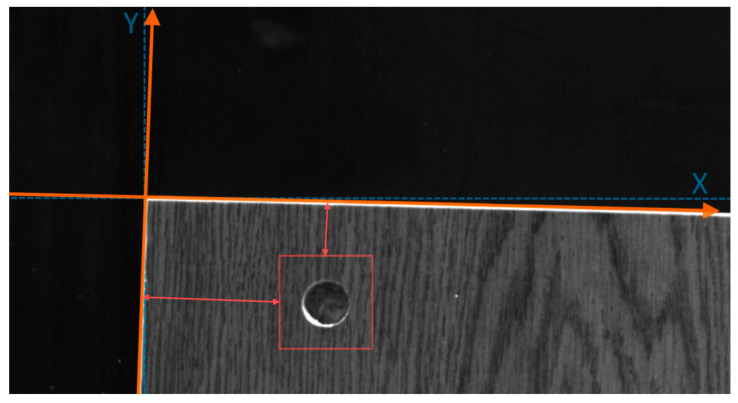
Furniture panel reference point and coordinate systems. The rotation of the object is evaluated and the coordinates system is turned accordingly. The hole drilling segmentation region is offset from the reference point by the given distance.

Drilled hole segmentation from the whole furniture panel can be a huge overhead for inspection timewise, taking into consideration huge image dimensions (the maximum size of the image is 6144 × 12384 pixels). Moreover, not all the panel’s area needs to be drilled. Therefore, knowing the place in the panel where drilling should be, only certain regions might be fed into the drilled hole segmentation neural network. Identified reference point in all particular model furniture panels can be assigned as coordinates system. From this point, all the drilling, according to the furniture template, needs to be located in the same places. The top-left point of the panel can be taken as the reference for the coordinate system. By extracting the panel from the conveyor belt and calculating the intersection between the top and the left side (panel’s edge) extrapolated lines, the coordinate system’s start could be found. Moreover, the part rotation can be evaluated from found edge lines. The idea of hole region search is shown in Figure 16.

The quality of the segmented drilled hole can be determined based on the *Dice* score or area differences between the template board and processed board. Further, the drilled hole position, according to its mass center point, can be evaluated. The drilled hole center point distance from the reference system start should be the same or diverge with the allowed error.

**Figure 17:**
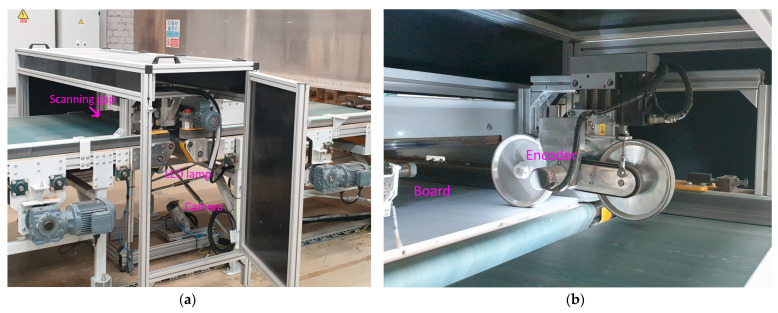
Image capture setup: (**a**) vision inspection box, (**b**) board scanning place. The camera is triggered with an encoder that is rotated by a roller that pushes down the furniture board.

Real inspection system implementation is given in Figure 17. A camera is placed near the ground and the LED light source is directed upwards (towards camera sensors direction). It is a different configuration than given in Figure 7. The camera (Figure 17a) is placed inside an additional metal safety cover with transparent windows that is blown by compressed air to remove the dust. Scanning is made through the gap between two conveyors. The camera is triggered by the encoder mounted on the roller that presses down the furniture board (Figure 17b), preventing it from shaking. Further, the rollers are covered with rubber to provide the grip with the board for precise movement detection (with encoder) that gives proper camera trigger. The whole image analysis system is covered to block outside light interference with separate analysis system lightning.

Visual wooden furniture panels surface inspection might take a different kind of algorithm than the proposed drilled holes segmentation method. However, the drilling regions should not be taken into consideration with regular (without drilling) areas in the furniture panel surface, or the drilled holes might be taken out from these regions and the rest of the region area could be considered as a regular surface and processed with surface defects detection algorithms.

In future work, we are considering utilizing a more advanced algorithm for surface defect inspection and edge inspection. The defects, such as faulty gluing and deficiency in paint coverage, appear in the lamination process. Moreover, surface damages might appear in any stage of manufacturing. Therefore, the inspection can be made from the same visual data.

## Figures and Tables

**Table 1:** Capturing set components.

Component	Model
Linear camera	raL6144-16 gm-Basler racer [55]
Camera optics	NIKON AF Nikkor 24 mm f/2.8D [56]
Encoder (on the motor)	Autonics E40S6-1500-3-T-24 [57]
Industrial LED lamp	EBAR-1125-WHI-7 TPL-Vision [58]

**Table 2:** Computer parameters.

Computer	CPU	RAM	GPU	OS
Desktop	AMD Ryzen 5 3600	16 GB	Nvidia 2070S	Windows 10
Laptop	Intel i5 8300H	16 GB	Nvidia 1050Ti	Windows 10

**Table 3:** Image processing methods performance results.

Method	Accuracy	Recall	Precision	IoU	Dice
*Sobel filter*	0.996943	0.919077	0.637585	0.580435	0.590472
*Laplace filter*	0.959769	0.931860	0.651680	0.607032	0.614552
*Canny edge detector*	0.934371	0.978507	0.693433	0.677103	0.685342

**Table 4:** Each model’s best-performing weights results.

CNN Architecture	Accuracy	Recall	Precision	IoU	Dice
*UNet*	0.999485	0.959081	0.958613	0.955272	0.944966
*UNet + SE*	0.998978	0.936343	0.978481	0.979132	0.953470
*UNet + CoordConv2D*	0.999390	0.961770	0.975089	0.973536	0.965975
*UNet + SE + CoordConv2D*	0.999102	0.949620	0.983831	0.980100	0.962330
*UNet + Res + ASPP*	0.999475	0.959433	0.973082	0.970765	0.961194
*UNet + Res + ASPP + SE*	0.999681	0.982027	0.977736	0.975958	0.978871
*UNet + Res + ASPP + CoordConv2D*	0.999548	0.967609	0.977881	0.974820	0.969476
*UNet + Res + ASPP + SE + CoordConv2D*	0.999414	0.962808	0.977196	0.974946	0.968346

## Data Availability

Contact MB Prorega (email: tadas@prorega.lt) for dataset inquiries.
